# Lethal and sub-lethal effects of low-temperature exposures on *Halyomorpha halys* (Hemiptera: Pentatomidae) adults before and after overwintering

**DOI:** 10.1038/s41598-020-72120-5

**Published:** 2020-09-17

**Authors:** Davide Scaccini, Luka Vanishvili, Paola Tirello, Vaughn M. Walton, Carlo Duso, Alberto Pozzebon

**Affiliations:** 1 Department of Agronomy Food Natural Resources Animals and Environment, University of Padova, 35020 Legnaro, Padua Italy; 2 Department of Horticulture, Oregon State University, Corvallis, OR USA

**Keywords:** Entomology, Invasive species

## Abstract

Cold winter temperatures can influence insects’ survival in temperate zones. Brown marmorated stink bug, *Halyomorpha halys* (Stål) overwinters as adults in natural and human-made structures. In this study, we characterized low temperature mortality rates of *H. halys* adults that were either entering (ENA) or exiting (EXA) overwintering microhabitats. We considered the effect of different duration of cold exposure on mortality. We determined the impact of insect nutritional status and weight on cold tolerance. We additionally evaluated the effects of cold exposure on *H. halys* adult longevity and fecundity. Mortality of ENA and EXA adults was determined for 6 h and 2 h periods at − 2.5 °C and 2.5 °C respectively. EXA adults displayed higher mortality rates compared to ENA individuals at the low-temperature regimes. *Halyomorpha halys* adult survival rate was higher when their nutrient index (EXA individuals only) and weight were high. Low-temperature exposure increased longevity, but reduced fecundity of ENA females. The data further highlight how extreme spring frost events can result in significantly increased mortality levels of *H. halys* adults.

## Introduction

Low temperature exposure during winter and extreme spring frost events can strongly contribute to a significant increase in mortality of overwintering insects. These events subsequently influence population size and damage risk to crops. Insects can overwinter through dormancy, which is an adaptive state characterized by suppressed development and metabolism. Diapause is a sub-type of dormancy characterized by metabolic suppression and arrested development and activity^1^. Induction of diapause in insects is largely mediated by changes in photoperiod and temperature^1–3^. In temperate climates, these environmental cues strongly influence and trigger key stink bug (Hemiptera: Pentatomidae) physiological processes, resulting in facultative diapause^4^. As an example, *Halyomorpha halys* (Stål) adults exposed to reduced photoperiod in fall exhibit increased searching behaviour for suitable overwintering locations including buildings and other structures that may provide more suitable microhabitats^5–9^.

*Halyomorpha halys* is an invasive species native to East Asia, and is now widespread in North America, Europe and South America^10^. This insect has been successfully intercepted at points-of-entry including New Zealand, thereby preventing known wild colonization^11,12^. *Halyomorpha halys* can attack and cause economic damage to more than 170 plant species. Damage is especially important on fresh fruit because of injury caused by the feeding activity and the transmission of pathogens^10,13–19^.

Suboptimal low winter temperature is a significantly important limiting factor, affecting population growth and range distribution, establishment and spread^20–22^. Climate conditions play a key role in the distribution of *H. halys* worldwide. Climate-based insect physiology models have demonstrated the future risk of pending *H. halys* invasion on important agricultural production regions in both the Northern and the Southern Hemispheres^23,24^.

Depending on the capability to survive intracellular ice formation, insects are classified as chill-intolerant (i.e., they die before freezing), freeze-intolerant (i.e., they live until they freeze) or freeze-tolerant (i.e., they are able to live after the formation of ice in the body)^21,25^. Recent studies on the supercooling point of *H. halys*, i.e., the temperature at which body fluids start to freeze, showed that this species should be considered chill-intolerant^26^.

Ice crystal formation may cause physical cell and tissue damage in an organism, and survival can be affected by osmotic stress, anoxia and damage in fat body cells^27–29^. Sub-lethal frost injury is most commonly observed in the failure to complete metamorphosis successfully^27^. Exposure to low temperatures may also have an impact on post-diapause survival, fecundity and behaviour, but these impacts can vary depending on the species even within a family, e.g. within Pentatomidae^4,30–33^.

Information on the effects of low temperatures on *H. halys* and identification of those critical for its survival can be used to model seasonal population dynamics and spatial distribution. In this study, we performed experiments aimed to: (1) characterize low-temperature mortality response of *H. halys* adults entering (ENA) and exiting dormancy (EXA); (2) quantify the effect of insect nutritional status and weight on tolerance to low temperature exposures; (3) evaluate the effects of exposure to low temperature on longevity and fecundity of surviving adults.

## Results

*Halyomorpha halys* adults used in this study experienced the following environmental conditions: 2.6 ± 0.1 °C (mean ± s.e.m.) in the coldest month (December), and 20.7 ± 0.2 °C in the warmest (May). The mean relative humidity was 77.2 ± 0.1%, and ranged between 67 and 84%.

**Figure 1:**
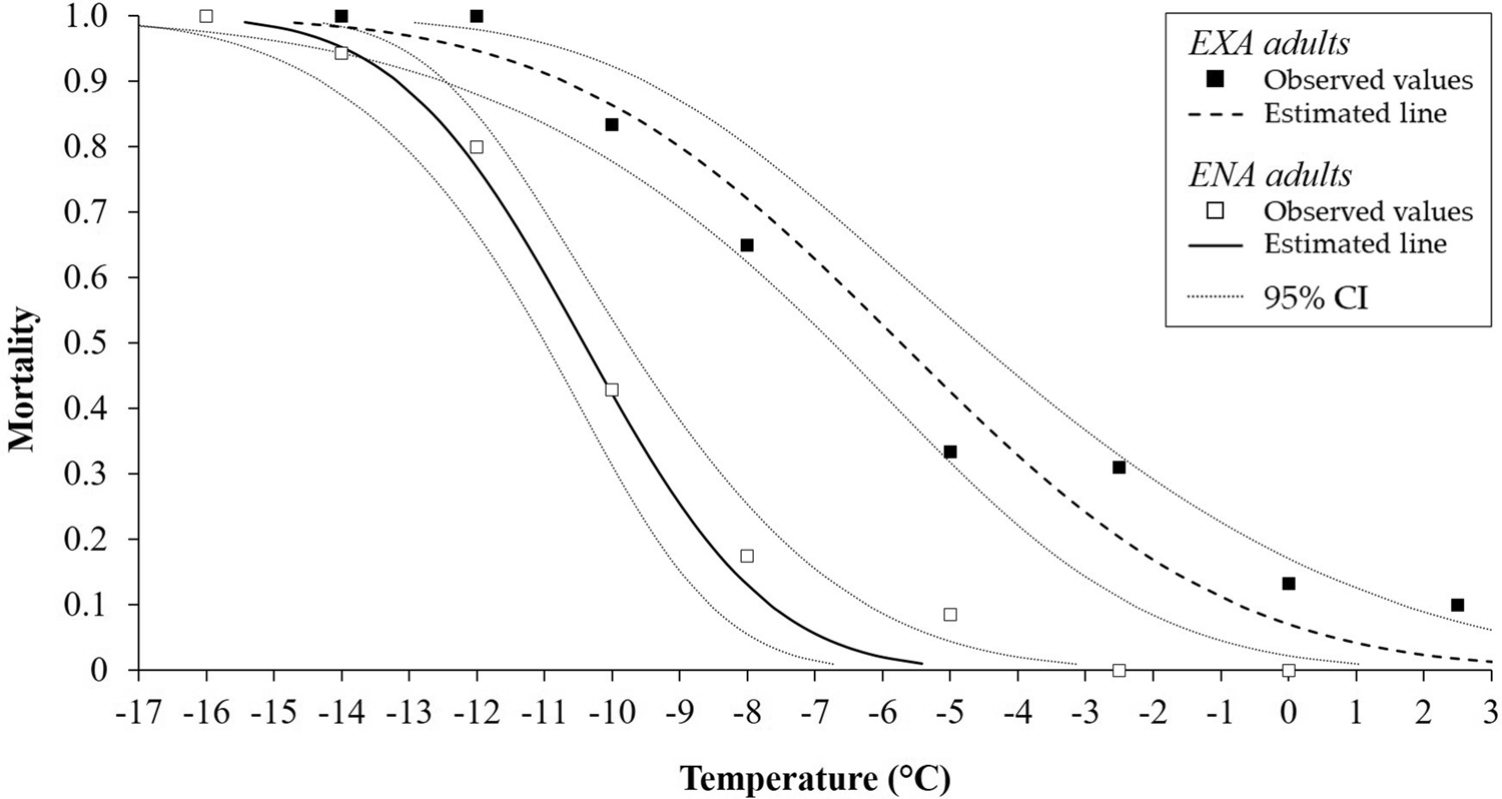
Mortality rate [observed and estimated values 95% confidence interval (CI)] of EXA and ENA *Halyomorpha halys* adults after exposure to controlled low temperatures for 2 h.

**Figure 2:**
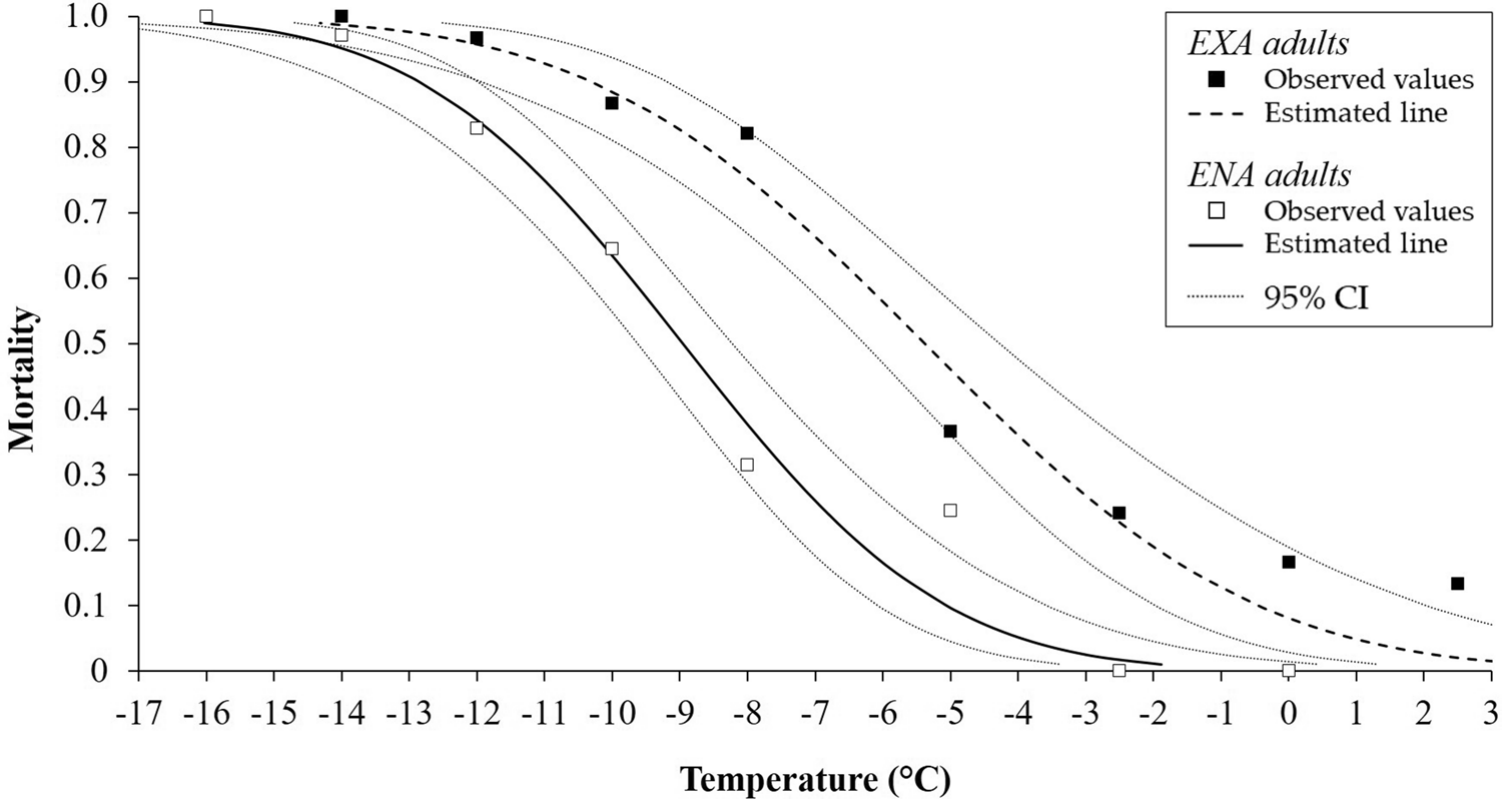
Mortality rate [observed and estimated values 95% confidence interval (CI)] of EXA and ENA *Halyomorpha halys* adults after exposure to controlled low temperatures for 4 h.

**Figure 3:**
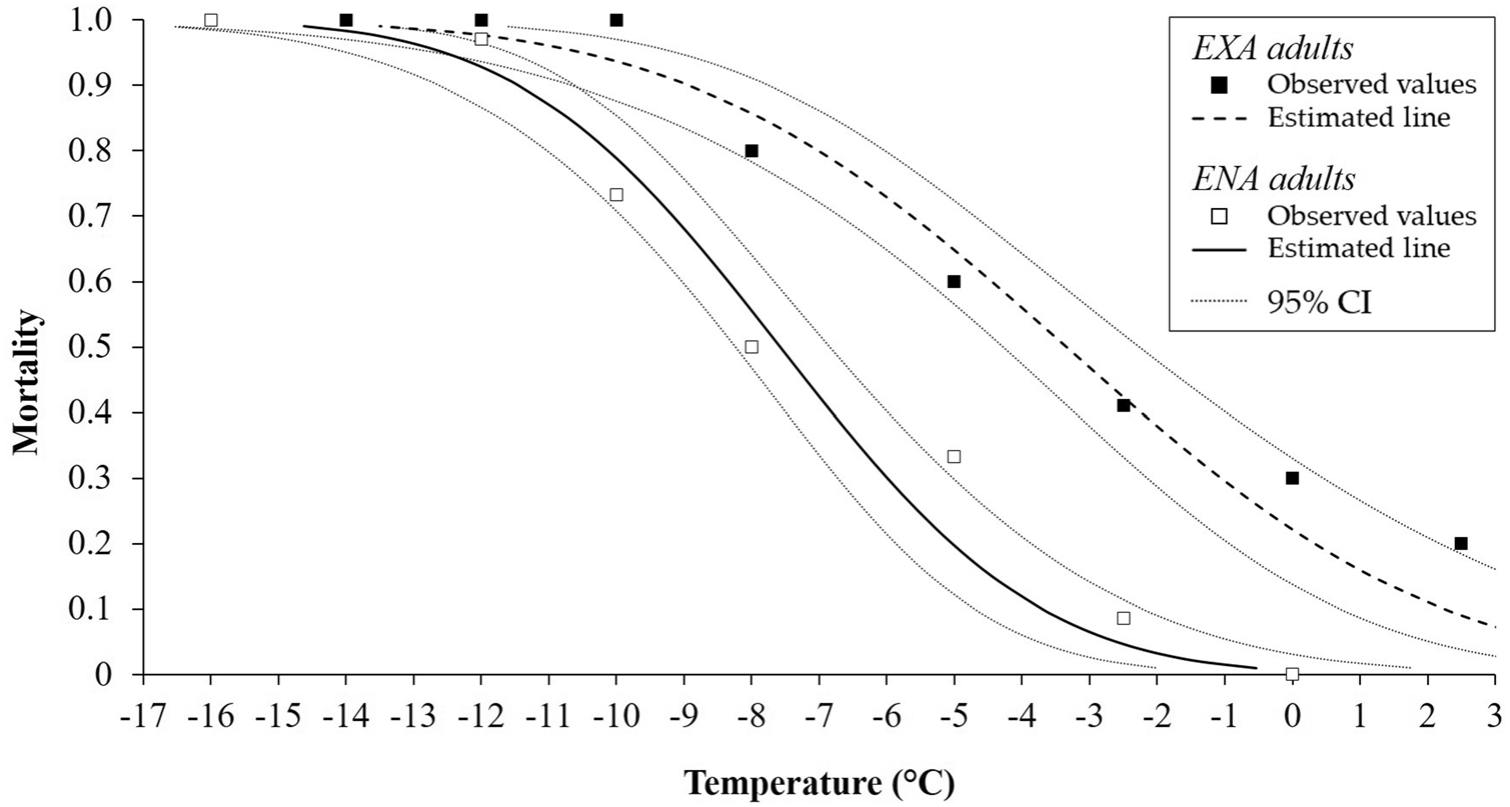
Mortality rate [observed and estimated values 95% confidence interval (CI)] of EXA and ENA *Halyomorpha halys* adults after exposure to controlled low temperatures for 6 h.

Low-temperature exposure was associated with relatively increased mortality levels of *H. halys* adults (Figs. 1, 2 and 3). Mortality of ENA adults was observed starting at − 5.0 °C for 2 and 4 h, and − 2.5 °C for 6 h (Figs. 1, 2 and 3). For EXA adults, mortality was observed at comparatively low levels at 2.5 °C for 2 h (10% of mortality rate). Mortality for EXA adults however increased to 20% at longer exposure time (Figs. 1, 2 and 3). For ENA adults, temperatures ranging from − 10.4 °C (2 h) to − 7.6 °C (6 h) resulted in 50% mortality (LT50). For EXA adults the LT50 was reached at temperatures ranging from − 5.7 °C (2 h) to − 3.3 °C (6 h; Table 1). The LT99 temperature regimes were found at − 16.0 °C (4 h) and − 14.6 °C (6 h) for ENA adults, and for EXA individuals they ranged from − 14.8 °C (2 h) to − 13.5 °C (6 h; Table 1). LT50s of EXA adults were always lower than those of ENA adults, while for LT99 differences emerged only at 4 h of exposure (Table 1).

**Table 1:** Lethal low temperatures LT50 and LT99 with 95% confidence interval (CI) and probit regression parameters for ENA and EXA *Halyomorpha halys* adults exposed for the three time periods (2, 4 and 6 h).

Stage of dormancy	Exposure time (h)	n	LT50 (°C)^a^		95% CI_LT50_ (°C)		LT99 (°C)^a^		95% CI_LT99_ (°C)		Intercept	s.e.m._Intercept_	Slope	s.e.m._Slope_	Χ^2^ ^b^	df
					Lower	Upper			Lower	Upper						
EXA	2	220	− 5.7331	a	− 6.7300	− 4.5745	− 14.7794	a	− 17.8056	− 12.9101	− 1.4743	0.2680	− 0.2572	0.0342	5.7715	6
ENA	2	280	− 10.4234	b	− 10.9874	− 9.7738	− 15.4251	a	− 17.3399	− 14.3061	− 4.8481	0.7488	− 0.4651	0.0675	4.7132	6
EXA	4	250	− 5.3761	a	− 6.2781	− 4.2754	− 14.3180	a	− 17.2263	− 12.5417	− 1.3987	0.2628	− 0.2602	0.0349	5.4501	6
ENA	4	300	− 8.9576	b	− 9.6253	− 8.2295	− 16.0277	b	− 18.0063	− 14.7096	− 2.9474	0.3705	− 0.3290	0.0370	10.2708	6
EXA	6	260	− 3.3432	a	− 4.2764	− 2.2652	− 13.4989	a	− 16.4673	− 11.5920	− 0.7658	0.1666	− 0.2291	0.0277	6.3769	6
ENA	6	300	− 7.5746	b	− 8.2368	− 6.8462	− 14.6200	a	− 16.5422	− 13.3306	− 2.5011	0.3218	− 0.3302	0.0366	8.1116	6

^a^LT values within a column for each exposure time paired with the same letter are not significantly different (α = 0.05) according to the Lethal Dose ratios method^64^.

^b^All χ^2^ values fit the model at α = 0.05.

**Figure 4:**
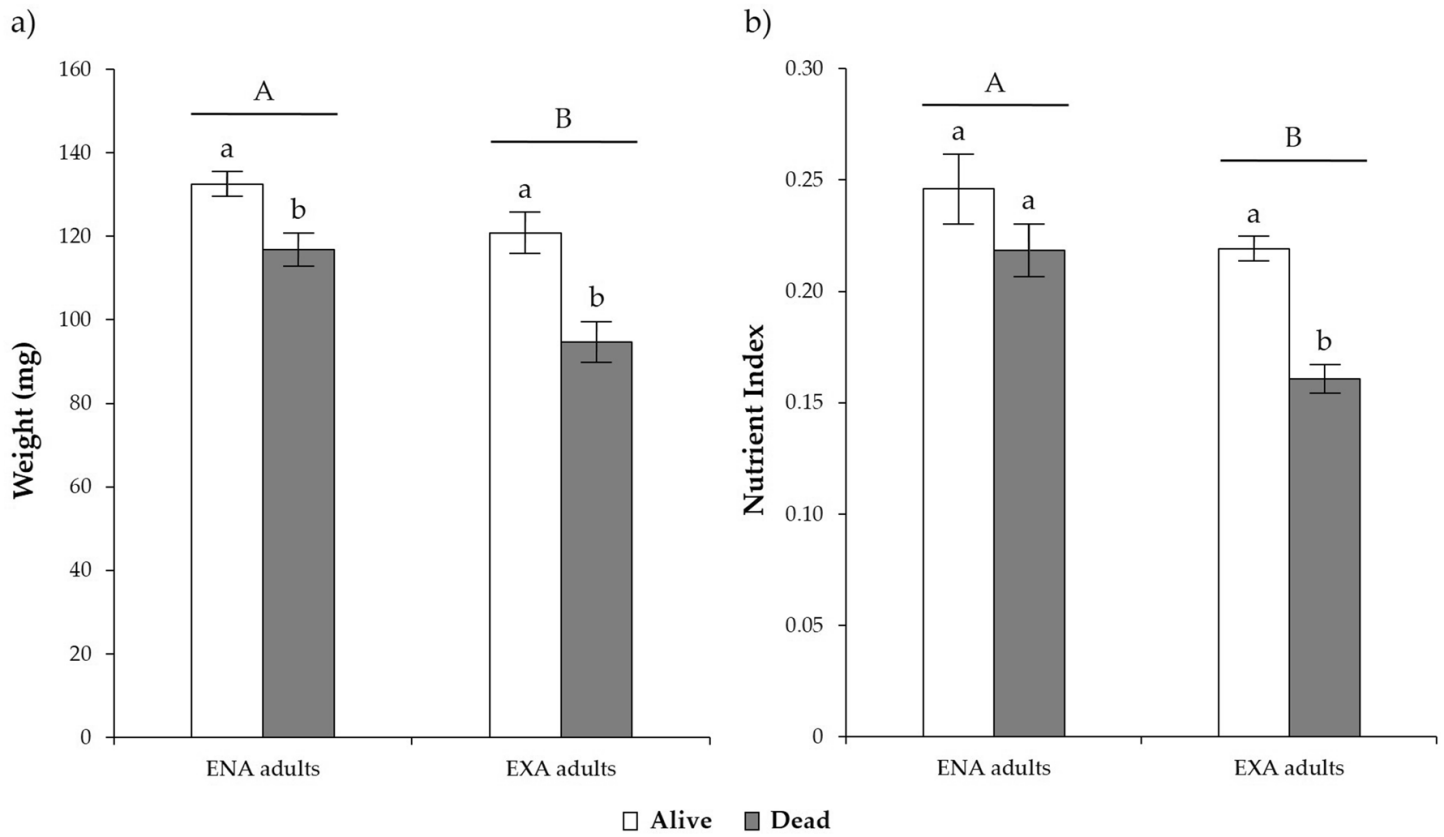
Mean (± s.e.m.) of *Halyomorpha halys* weight (**a**) and nutrient index (**b**) exposed to low temperatures in relation to their status (dead or alive) and the stage of dormancy (EXA or ENA). Different letters indicate significant differences at the Tukey–Kramer test (α = 0.05). Capital letters: comparisons between EXA and ENA adults; lowercase letters: comparisons between alive and dead adults.

The weight and nutrient index were significantly higher for ENA compared to EXA individuals (Table 2; Fig. 4a,b). ENA and EXA adults that survived low temperature exposures were characterized by a high body weight (Table 2; Fig. 4a), and the surviving EXA individuals had a higher nutrient index than those that died (Table 2; Fig. 4b).

**Table 2:** Statistics of GLMM models (α = 0.05) on the weight and nutrient index measured on *Halyomorpha halys* exposed to low temperatures.

Source of variation	Weight			Nutrient index		
	F value	df	*P* value	F value	df	*P* value
Status (dead or alive)	**5.54**	**1, 194**	**0.0196**	**23.23**	**1, 194**	**< 0.0001**
Stage of dormancy (EXA or ENA)	**8.64**	**1, 194**	**0.0037**	**25.51**	**1, 194**	** < 0.0001**
Stage of dormancy*Status	1.18	1, 194	0.2795	**4.28**	**1, 194**	**0.0399**
Time	0.73	2, 194	0.4843	0.40	2, 194	0.6729
Time*Status	0.59	2, 194	0.5526	0.92	2, 194	0.4000
Time*Stage of dormancy	0.09	2, 194	0.9142	0.01	2, 194	0.9882
Time*Stage of dormancy*Status	0.17	2, 194	0.8446	0.05	2, 194	0.9553

Statistically significant sources of variation are reported in bold.

**Figure 5:**
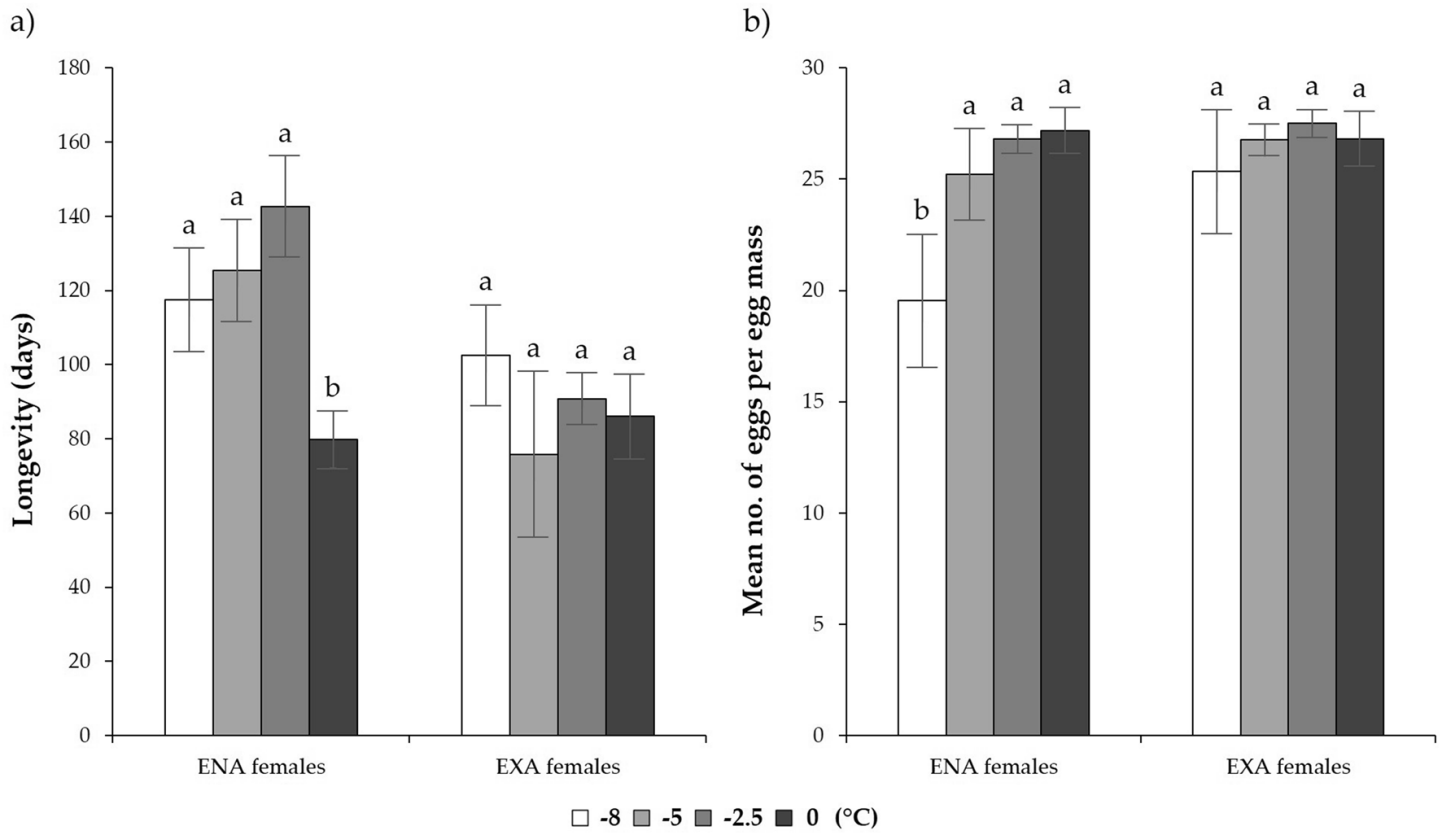
Mean (± s.e.m.) of *Halyomorpha halys* female longevity (**a**) and mean (± s.e.m.) number of eggs laid per egg mass (**b**) at different low-temperature exposures, in relation to their stage of dormancy. Different letters indicate significant differences at the Tukey–Kramer test (α = 0.05).

Low temperature exposure significantly affected the longevity of ENA adults. Exposure temperatures below 0 °C were associated with increased longevity for these individuals (Table 3; Fig. 5a). However, for EXA adults we observed no differences in longevity following low temperature exposures. ENA individuals exposed to − 8.0 °C showed a lower number of eggs per egg mass compared to other treatments within this group. However, there were no significant differences in fecundity of EXA females (Table 4; Fig. 5b).

**Table 3:** Statistics of GLMM models (α = 0.05) on *Halyomorpha halys* female pre-oviposition period and longevity.

Stage of dormancy	Effect	Pre-oviposition period			Longevity		
		F value	df	*P* value	F value	df	*P* value
ENA	Temperature	0.47	3, 43	0.7030	**6.20**	**3, 70**	**0.0008**
ENA	Time	0.62	2, 43	0.5419	0.78	2, 70	0.4626
ENA	Temperature*Time	1.26	6, 43	0.2939	0.51	6, 70	0.8012
EXA	Temperature	1.28	4, 27	0.3010	0.79	4, 30	0.5431
EXA	Time	0.43	2, 27	0.6573	0.63	2, 30	0.5372
EXA	Temperature*Time	1.89	8, 27	0.1031	1.33	8, 30	0.2684

Data were analysed separately by the stage of dormancy (EXA vs. ENA). Statistically significant effects are reported in bold.

**Table 4:** Statistics of GLMM models (α = 0.05) on *Halyomorpha halys* fecundity (i.e., number of egg masses laid, the mean number of eggs per egg mass, and the total number of laid eggs per female) and egg hatching rate.

Stage of dormancy	Effect	No. of egg masses			Mean no. of eggs per egg mass			Total no. of eggs			% of hatching		
		F value	df	*P* value	F value	df	*P* value	F value	df	*P* value	F value	df	*P* value
ENA	Temperature	1.78	3, 47	0.1642	**3.22**	**3, 41**	**0.0325**	2.19	3, 41	0.1043	0.95	3, 47	0.4241
ENA	Time	1.84	2, 47	0.1702	1.79	2, 41	0.1802	0.82	2, 41	0.4489	1.70	2, 47	0.1941
ENA	Temperature*Time	0.94	6, 47	0.4783	1.13	6, 41	0.3628	1.25	6, 41	0.3028	1.58	6, 47	0.1737
EXA	Temperature	0.41	4, 30	0.7975	0.87	4, 30	0.4959	0.99	4, 31	0.4293	0.54	4, 31	0.7068
EXA	Time	0.34	2, 30	0.7166	0.47	2, 30	0.6314	0.65	2, 31	0.5291	0.64	2, 31	0.5322
EXA	Temperature*Time	0.71	8, 30	0.6828	0.44	8, 30	0.8847	0.70	8, 31	0.6856	1.93	8, 31	0.0900

Data were analysed separately by the stage of dormancy (EXA vs. ENA). Statistically significant effects are reported in bold.

## Discussion

Results from controlled low-temperature/time regimes provide valuable information on *H. halys* adult mortality. High mortality levels (i.e., 99%) were obtained at temperatures lower than − 13.5 °C, for periods that persisted for at least 6 h. Our data are consistent with previous studies on *H. halys* supercooling point levels, showing a range of − 17.1 to − 13.9 °C^26^.

The results obtained here show that there are differences in cold tolerance between insects entering and exiting dormancy (i.e., ENA vs. EXA). At exposures of 2 h, the LT50 for ENA adults was − 10.4 °C, while it was − 5.7 °C for EXA individuals. At exposures of 6 h, the LT50 for ENA was − 7.6 °C, while it was − 3.3 °C for EXA individuals. This is not surprising, since cold tolerance is enhanced in diapausing *H. halys* adults compared to non-diapausing individuals^34^. It is known that insects entering diapause generally reduce water loss by converting carbohydrates into fats^29,35^, and this change ultimately results in increased desiccation resistance and energy conservation^36^. Insect metabolism is sugar-mediated at very low and sub-freezing temperatures. Lipids are typically used as an energy source in autumn and spring, thereby enhancing winter survival. It is believed that tissues that have reduced liquid and increased sugar content may have similar qualities as antifreeze during these periods^37^. It is possible that low nutrient levels in *H. halys* adults may impact susceptibility to suboptimal low temperatures before, during and after dormancy^38^.

The ability of insects to survive low temperatures, called cold-hardiness^39,40^, can change after rapid cold exposures^41,42^. This is a necessary and essential trait for the survival of populations when exposed to temperatures below 0 °C during cold periods of the year^43^. The data obtained here however highlight that the dormancy mechanisms used to cope with extreme temperatures are not effective at temperatures close to supercooling points for *H. halys*. These findings have clear implications in forecasting the mortality of *H. halys* populations after exposure to low winter temperatures or spring frost. Mortality curves, obtained from interpolated data on ENA individuals, suggest that overwintering *H. halys* mortality is observed at temperatures below − 5.0 °C for 2 h. Previous research on *H. halys* adults exposed to both episodic and gradual cold shock events suggest that post-diapause survival was less than 25% if individuals were exposed to − 6.0 °C. The survival reached 40–50% when the adults were exposed to temperatures ranging from − 2.0 to 2.0 °C^33^.

Data on EXA individuals suggest that mortality can occur when adults are exposed to 3.0 °C for 2 h. These findings should be considered in the context of climate change. Increasing temperatures are likely associated with warmer winter temperatures and decreased mortality of *H. halys*. Warmer spring temperatures can alternatively result in an advance of the seasonal phenology of both plants and insects, and this physiological change can potentially increase the risk of spring frost damage not only to plants, but also insects^4,32,44–50^. These changes can significantly impact pest infestation levels during the growing season. Frost occurrence has both direct (i.e., mortality by low-temperature exposures) and indirect effects (e.g., loss of food sources) on animal and plant distribution, growth and reproduction^51^. *Halyomorpha halys* adult emergence from overwintering sites can start when ambient temperature increase to above 10.0 °C. Flight activity generally occurs starting at 15.0 °C or more^52–54^. Thus, when warm weather events happen unseasonably early in spring, *H. halys* adults can similarly emerge early from their overwintering sites. Later spring frost at or below 0 °C will undoubtedly result in increased mortality in *H. halys* adults, especially if these follow-up frost events persist for long periods.

The weight and nutrient index of *H. halys* could be an indicator of low temperature tolerance. The lengthy overwintering period is a stressor, which results in a decline in the nutritional levels of *H. halys* adults^38,55^. Such lower nutrient index is undoubtedly related to lowered survival rates of post-diapause *H. halys* adults in this study^38,55^. It is possible that *H. halys* adults can counteract this phenomenon by feeding on suitable food source as they exit from dormancy. Altogether, it is clear from this and previous studies that nutrient reserves play a key role in the post-diapause survival of *H. halys* adults^38^. Differences in the susceptibility to low temperatures are affected by sex (male or female), and observed clearly when considering their supercooling point^26^.

Low-temperature exposure can determine sub-lethal effects on surviving *H. halys*, in particular in ENA adults, which showed an increase in longevity but a reduction in the number of eggs laid per egg mass. Studies on other stink bugs, such as *Nezara viridula* (L.), showed increased survival and fecundity after the exposure to mild temperatures during winter–early summer^32,47^. Similarly, diapausing *H. halys* adults exposed to temperatures below − 4.0 °C displayed an increased fecundity levels after diapause^56^. Differences between studies may depend on the methodological approach used^57–59^, and it is clear that additional work is required to clarify the sub-lethal effects of low temperatures on these pests.

Since the *H. halys* adults collected for this study came from buildings when they were searching for overwintering shelters, they may differ in age, possibly influencing their lifespan. Additional factors possibly impacting lifespan include mating status, as it is known in Hemiptera^60^ and Coleoptera, even though in many cases the reproduction cost in females can be low, in particular when food sources are not scarce^61^.

In conclusion, our results shed light on the impact of low temperatures on *H. halys* mortality. These results can be used to forecast the survival rate of this pest after winter, with implications for population abundance modelling. These data can also be taken into account to predict the geographic distribution pattern of *H. hays* under different climate change scenarios.

## Methods

### Insects

*Halyomorpha halys* searching for overwintering sites (ENA adults) were collected on building walls in Legnaro, Italy (45.3449°N, 11.9562°E), in September–October 2017 and 2018. In the collection area, temperature and relative humidity were monitored using data loggers (HOBO U23 Pro v2 Temperature/Relative Humidity Data Logger, Onset Computing, Bourne, MA, USA) as well as nearby weather stations. We placed more than 1,300 adults in five artificial overwintering units (at least 250 per unit). Each overwintering unit consisted of a wooden cage (34 × 19 × 10 cm) filled with cardboard and paper pieces as shelter (abt. 20 × 20 cm). A slit (34 × 1 cm) was present along one side of the wooden cage to allow adults to exit from overwintering units in spring. Each wooden cage was placed inside a transparent plastic box (50 × 35 × 15 cm; IKEA, Delft, Netherlands). The overwintering units were maintained under shaded outdoor conditions during the two winters and were monitored daily to detect insects exiting overwintering (EXA adults). Temperature and relative humidity within overwintering units were monitored using data loggers (HOBO U23 Pro v2 Temperature/Relative Humidity Data Logger, Onset Computing, Bourne, MA, USA). Insects from overwintering units were collected in April–May as soon as they were found outside the wooden boxes and were found moving in the plastic boxes. Adults that left overwintering shelters before April were not considered in this study because they were only a few individuals. Insect dissection performed under a stereomicroscope (Stemi 508, Carl Zeiss Microscopy GmbH, Jena, Germany) confirmed that EXA and ENA females were in the ‘one immature oocyte per ovariole’ rank as described by Nielsen et al.^62^, with undeveloped ovaries and thin oviducts and spermatheca.

### Temperature-mortality curves

Temperature-mortality curves were calculated to characterize the response in terms of mortality of *H. halys* adults after exposure to low temperatures for different exposure times. Low temperatures used in the experiment were 2.5 (only for EXA), 0, − 2.5, − 5.0, − 8.0, − 10.0, − 12.0, − 14.0, or − 16.0 °C (only for ENA) for 2, 4 or 6 h as exposure times. Insects were individually placed in a glass vial of 7-ml volume, closed by a cotton swab to allow gas exchange (available volume: about 5.5 ml after placing the cotton swab). To perform low-temperature exposures, vials containing insect were immersed in thermostatic liquid (Kryo 30, LAUDA Dr. R. Wobser GMBH & Co. KG, Lauda-Königshofen, Germany) of a cooling thermostat (Alpha, RA 12, LAUDA Dr. R. Wobser GMBH & Co. KG, Lauda-Königshofen, Germany) set at a static pre-determined experimental temperature. Vials were placed in the water bath immediately after the *H. halys* adults were inserted therein. Cooling to the set temperature was − 6.9 ± 0.7 °C min^−1^ (mean ± s.e.m.), and the set temperature was reached in less than 5 min. A thermocouple (RoHS, 4-Channel Digital Thermometer Thermocouple Sensor, Omega Engineering, Norwalk, CT, USA) was used to check the temperature inside vials. During the exposure, the relative humidity within vials ranged from 46 to 54% (Digital LCD Dual Display Thermometer—Hygrometer with Probe, Guangzhou Juanjuan Electronic Technology Co., Ltd., Guangzhou, China). At least 20 EXA or 35 ENA, up to 45, adults for each temperature/time combination were tested. Batches of five insects were individually exposed at the same time and were considered as treatment replicates. An equal number of females and males was used in the experiments. After low-temperature exposure, adults were removed from vials and transferred to bug dorms (30 × 30 × 30 cm; BugDorm-1, MegaView Science Co., Ltd., Taiwan), grouped for the same exposure temperature/time combination, kept at room temperature and checked after 24 h for mortality assessment. Mortality was assessed through the inspection of each group of adults. Individuals were considered dead if their appendages did not move when prodded with a pin. Control treatments were performed at room temperature (23.0 ± 1.0 °C).

### Effects of low temperature on longevity, fecundity and egg hatch of surviving females

To study the sub-lethal effects on *H. halys*, survived insects exposed to low temperatures were paired males and females from the same exposure treatment and placed in bug dorm under controlled conditions at a temperature of 23.0 ± 1.0 °C, 46–54% RH and a photoperiod of 16:8 (L:D). Four to 32 *H. halys* EXA male–female pairs and 11 to 15 ENA pairs were obtained from each treatment. All the insects were reared in the bug dorms with carrots (*Daucus carota* L.), green beans (*Phaseolus vulgaris* L.), and unshelled sunflower seeds (*Helianthus annuus* L.), replaced every week (carrots and green beans) or when moulding/rotting or becoming too dry (sunflower seeds). Water was supplied ad libitum on a cotton swab. Rearing boxes were checked every day for insect survival and presence of egg masses. Laid egg masses were removed and placed separately in a plastic box (14 × 4 × 11 cm), with cotton swabs with water and kept at room temperature with 46–54% RH and a photoperiod of 16:8 (L:D) until hatching. Hatching rate (i.e., number of hatched nymphs/number of eggs in the egg mass), was evaluated for each egg mass. The collected data were used for the calculation of female longevity after treatment (number of days from the end of the treatment and the death of the female), length of the pre-oviposition period (number of days from the end of the treatment and the first egg mass found), number of egg masses, the mean number of eggs per egg mass and the total number of eggs.

### Data analysis

Data on the lethal effect of low temperatures were analysed with a probit regression using the PROBIT procedure of SAS (ver. 9.4)^63^, interpolating the observed data to mortality curves. For any exposure time, lethal temperatures causing 50% (LT50^39^) and 99% (LT99) of adults’ mortality were estimated. Comparisons were made between the two groups of adults (i.e., EXA and ENA) for the LT50 and LT99 following the Lethal Dose ratios method (α = 0.05), which is based on their 95% confidence limits and depends on the intercepts and the slopes of the probit lines, also considering the variance–covariance matrices^64^.

For a subset of insects (480 ENA and 300 EXA adults) exposed to low temperatures as described before, the nutrient index was calculated as weight (mg)/prothorax width (mm)^3^. This index is considered a proxy of nutritional levels of an insect, and it was previously used to evaluate the nutritional level of *H. halys* individuals^38,55,65,66^. Differences on weight and nutrient index were considered between dead and alive insects after exposure to low temperatures for 2, 4 or 6 h, through a General Linear Mixed Model with the MIXED procedure of SAS (ver. 9.4)^63^ and an F-test (α = 0.05), followed by a Tukey–Kramer test (α = 0.05). The status of the insect (i.e., dead or alive), stage of dormancy (i.e., EXA or ENA), time of exposure (i.e., 2, 4 or 6 h) and their interactions were considered as sources of variation. Data were checked for model assumptions prior to the analysis, and untransformed data were used.

Finally, the effects of low-temperature exposure on surviving females were evaluated through a Generalized Linear Mixed Models with the procedure GLIMMIX of SAS (ver. 9.4)^63^. The effect of temperature application, exposure time and their interaction were considered as independent variables. Their effect was tested with an F-test (α = 0.05) and means were separated using a Tukey–Kramer test (α = 0.05) on least-square means. Data of survived females exposed to − 12.0 °C and − 10.0 °C were not included in the analysis due to the low number of survivals. Data were transformed in log (x + 1) to meet model assumptions.

### Ethical approval and informed consent

This study did not include research on vertebrates or humans. All studies were carried out in accordance to the highest relevant ethical, scientific, and institutional guidelines.

## Data Availability

The datasets generated during and/or analysed during the current study are available from the corresponding author on reasonable request.
